# 

**Published:** 1884-10

**Authors:** 


### Contents—October.

ORIGINAL COMMUNICATIONS:

Herbst's New Method of Filling Teeth. W. D. Miller........ 541
A Visit to the Dentist. One Who Has Been There........ 544
Amalgam. S. C. G. Watkins........ 552
A Case in Practice. C. H. Eccleston........ 557
A Wandering Supernumerary Tooth. E. S. Talbot........ 558
Unusual Case of Cleft Palate........ 559

REPORTS OF SOCIETY MEETINGS:

American Dental Association........ 560
American Medical Association........ 565
Pennsylvania State Dental Society........ 568
Illinois State Dental Society........ 573
New Jersey State Dental Society........ 576

EDITORIAL:

October........ 585
The Earthy Phosphates........ 587
Text Books........ 588
A Provoking Blunder........ 589

CURRENT NEWS AND OPINION:

The Tooth Crown Patents........ 589
Pediatric Aphorisms........ 590
Analyses of Beef Peptonoids........ 592
Dentists' Benevolent Association........ 593
Transactions of the New York State Dental Society........ 594
Dental Education in Germany........ 594
New England Dental Society........ 595
Death of Cohnheim........ 595
Fifth and Sixth District Societies........ 595
Assistant Wanted........ 595
Seventh and Eighth District Dental Societies........ 595
Askings and Answers........ 596

